# Patient-Level Prediction of Cardio-Cerebrovascular Events in Hypertension Using Nationwide Claims Data

**DOI:** 10.2196/11757

**Published:** 2019-02-15

**Authors:** Jaram Park, Jeong-Whun Kim, Borim Ryu, Eunyoung Heo, Se Young Jung, Sooyoung Yoo

**Affiliations:** 1 Office of eHealth Research and Business Seoul National University Bundang Hospital Seongnam Republic of Korea; 2 Department of Otorhinolaryngology Seoul National University Bundang Hospital Seongnam Republic of Korea; 3 Department of Otorhinolaryngology Seoul National University College of Medicine Seoul Republic of Korea; 4 Department of Family Medicine Seoul National University Bundang Hospital Seongnam Republic of Korea

**Keywords:** health risk appraisal, risk, hypertension, chronic disease, clustering and classification, decision support systems

## Abstract

**Background:**

Prevention and management of chronic diseases are the main goals of national health maintenance programs. Previously widely used screening tools, such as Health Risk Appraisal, are restricted in their achievement this goal due to their limitations, such as static characteristics, accessibility, and generalizability. Hypertension is one of the most important chronic diseases requiring management via the nationwide health maintenance program, and health care providers should inform patients about their risks of a complication caused by hypertension.

**Objective:**

Our goal was to develop and compare machine learning models predicting high-risk vascular diseases for hypertensive patients so that they can manage their blood pressure based on their risk level.

**Methods:**

We used a 12-year longitudinal dataset of the nationwide sample cohort, which contains the data of 514,866 patients and allows tracking of patients’ medical history across all health care providers in Korea (N=51,920). To ensure the generalizability of our models, we conducted an external validation using another national sample cohort dataset, comprising one million different patients, published by the National Health Insurance Service. From each dataset, we obtained the data of 74,535 and 59,738 patients with essential hypertension and developed machine learning models for predicting cardiovascular and cerebrovascular events. Six machine learning models were developed and compared for evaluating performances based on validation metrics.

**Results:**

Machine learning algorithms enabled us to detect high-risk patients based on their medical history. The long short-term memory-based algorithm outperformed in the within test (F1-score=.772, external test F1-score=.613), and the random forest-based algorithm of risk prediction showed better performance over other machine learning algorithms concerning generalization (within test F1-score=.757, external test F1-score=.705). Concerning the number of features, in the within test, the long short-term memory-based algorithms outperformed regardless of the number of features. However, in the external test, the random forest-based algorithm was the best, irrespective of the number of features it encountered.

**Conclusions:**

We developed and compared machine learning models predicting high-risk vascular diseases in hypertensive patients so that they may manage their blood pressure based on their risk level. By relying on the prediction model, a government can predict high-risk patients at the nationwide level and establish health care policies in advance.

## Introduction

Nationwide health maintenance programs are aimed at the prevention of chronic diseases. South Korea has a single-payer national health insurance system in which all health care providers must participate to claim for their medical expenses [1]. In this efficient health care system, the Korean government provides a nationwide health maintenance program to all national health insurance members aged 40 years and above on a biennial basis [2].

Hypertension is one of the most important chronic diseases requiring management via the nationwide health maintenance program because the burden of this condition is enormous. Approximately 10% of the total medical expenditure is associated with hypertension and its attendant complications, resulting in high economic costs [3]. In particular, East Asia and the Pacific region have the highest absolute burden of 439 million hypertensive patients [4]. Moreover, increased blood pressure leads to 9.4 million deaths associated with ischemic heart disease, stroke, and heart failure. However, among the general population of hypertensive patients, just 46.5% were found aware of their existing condition, 36.9% received treatment, and only 13.8% actively controlled their blood pressure [5]. Hypertension and its complications are attributed to modifiable risk factors, such as high salt diets, physical inactivity, and obesity. Therefore, health care providers should inform patients about their risks of complications caused by hypertension, so that they can improve the modifiable risk factors [6,7].

Health risk appraisal (HRA) is one of the most widely used screening tools for increasing both, the awareness and treatment levels of hypertension [8,9]. From 2009, it has been provided for all patients included in the national health maintenance program in South Korea to outline the importance of controlling high blood pressure. However, there are some limitations in using the HRA for predictive purposes. First, the predictability of cardio-cerebrovascular events is not very reliable. Second, it is based on a static statistical model that is not dynamically improvable on a regular basis. Finally, HRA is usable only when a patient is included in the health maintenance program. Due to these limitations, patients with hypertension tend to overlook their risks of developing cardio-cerebrovascular complications, in turn lowering the treatment rate of hypertension. In this study, we aimed to develop and compare machine learning models predicting high-risk vascular diseases for hypertensive patients, so that they can manage their blood pressure based on their risk level.

To develop these models, we used the longitudinal dataset of the nationwide sample cohort, which allowed tracking patients’ medical history across all health care providers in Korea (N=51,920) spanning 12 years. Furthermore, to ensure the generalizability of our models [10-12], we conducted an external validation using another national sample cohort dataset, comprised of different patients, published by National Health Insurance Service (NHIS).

## Methods

### Data Description

Based on the mandatory social insurance system, the Korean National Health Insurance Service (NHIS) has achieved universal coverage of the population since the mid-1970s [1]. The NHIS is a single-insurer system, and the system has paid health care providers based on fee-for-service. Therefore, almost all health care data are centralized in the large-scale database of NHIS [13]. In this study, we used two distinct datasets published independently by NHIS. The first dataset was used to develop and test machine learning models, and the second was used to conduct external validation of developed models.

To develop and test machine learning models, we used a sample cohort of national health check-up programs [2,14]. The NHIS provides a biennial health check-up program to all national health insurance members over 40 years of age free of charge. The dataset contains health records of a total of 514,866 patients, randomly selected from the health insurance members who have been served the health check-up program. For external validation, we used a national sample cohort dataset, which includes the data of one million patients who are randomly selected comprising 2.2% of the total Korean population in 2002. The dataset includes health records of patients from infants to elderly people over 85 years old. Both datasets contain patients’ social and economic qualification variables, the status of medical resource utilization, statement, details of treatment, type of disease, and details of prescription, and the status of the clinic [13,14]. The detailed information of the variables in both datasets is described in Multimedia Appendix 1. With the benefits of a 12-year longitudinal dataset reflecting a nationwide sample cohort, we were able to track each patient’s medical history from all types of health care providers (N=51,920), including a tertiary hospital (number of beds ≥300), general hospitals (number of beds ≥30), and clinics. To protect patient privacy, the personal information and clinical institution information were deidentified. The statistics of the NHIS datasets used for building models are presented in Table 1.

The study was approved by the Seoul National University Bundang Hospital Institutional Review Board (B-1512/326-102).

**Table 1:** Statistics of National Health Insurance Service dataset (2002-2013). The precise percentage of the numbers in this table cannot be provided because the official total numbers are unavailable. However, we believe that each dataset contains almost all the medical records of the sampled patients, since South Korea has a mandatory social insurance system that meets the universal coverage of the population and medical institutions.

Description	Health check-up cohort (n)	National sample cohort (n)
Hospitals	51,920	52,483
Patients	514,866	1,113,656
Prescriptions	83,935,395	83,935,395
Visits	96,534,359	119,362,188
Diagnostic codes (full code name)	17,385	19,626
Diagnostic codes (first 3-digits)	2160	2319
Annual patient visits, mean	15.6	8.9
Diagnostic codes/visit, mean	2.4	2.5
Drugs/prescription, mean	4.4	4.4

### Study Population Definition

We focused on patients with essential hypertension and developed models to predict cardio-cerebrovascular events. Therefore, we identified patients with confirmed essential hypertension and cardio-cerebrovascular events based on the results of previous research [15,16]. Patients with hypertension were defined as the subjects newly diagnosed with essential hypertension (International Classification of Diseases, Tenth Revision ICD-10: I10, I100, or I109) and newly treated with at least one Anatomical Therapeutic Chemical (ATC) code that is related to hypertension between June 2004 and December 2008 (the ATC codes used in this study are presented in Multimedia Appendix 2). Patients with cardio-cerebrovascular were defined as the subjects newly diagnosed with ischemic heart disease (ICD-10: I20-I25), cerebrovascular diseases (ICD-10: G45, I60-I64, I65-I69), or chronic heart failure (ICD-10: I42, I50), or newly treated with at least one ATC code that is related to cardio-cerebrovascular medication (the ATC codes are presented in Multimedia Appendix 2).

We excluded patients with any previous record of antihypertensive medication, essential hypertension, ischemic heart disease (ICD-10: I20-I25), cerebrovascular diseases (ICD-10: G45, I60-I64, I65-I69), or chronic heart failure (ICD-10: I42, I50) during the washout period between May 2003 and May 2004.

### Input Features and Algorithms

**Figure 1:**
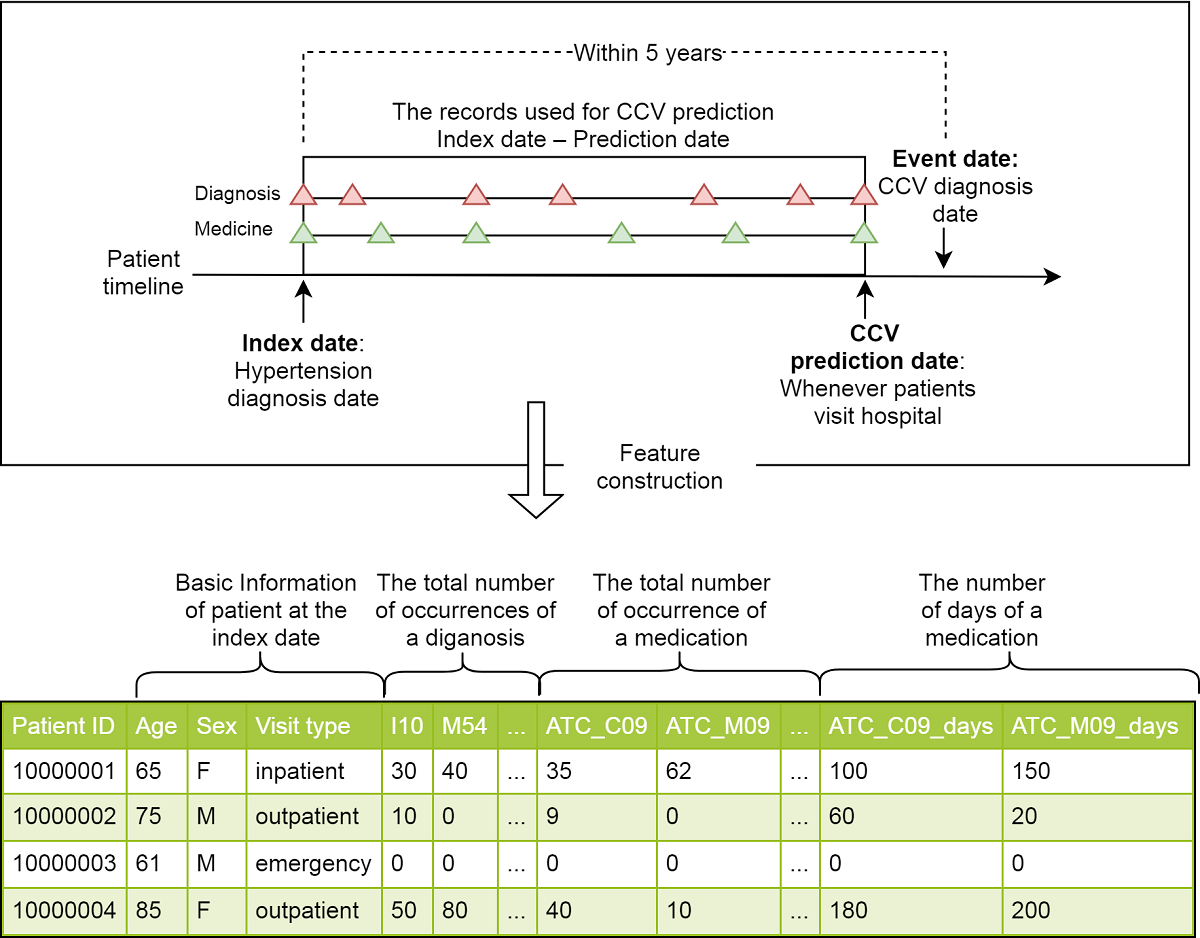
Feature matrix construction. CCV: cardio-cerebrovascular.

The index date of each patient was defined as the time point of the first hypertension diagnosis or the time point of the first medication records related to hypertension. The event date of each patient was defined as the time point of the first cardio-cerebrovascular diagnosis within five years (see Figure 1). The prediction model application scenario was that when a patient visits a hospital, the models predict current or near-term risks which we defined as the duration from current/today to the next hospital visit (one month on average) high-risk vascular disease events in a patient to enable patients to manage blood pressure immediately. We limited the prediction periods to five years from the first hypertension diagnosis. Therefore, the algorithms predict current high-risk vascular disease events with medical records from a maximum of five years ago.

Of all medical records, we selected the following main features. First, the basic information of the patient, including age, gender, and hospital visit type (inpatient, outpatient, emergency) at the index date (n=3). Second, all diagnosis records from the index date to the event date (n=1252). All diagnosis records (ie, ICD-10 codes) were grouped by the first 3 digits, which comprise the main disease category. Each dimension represents the total number of occurrences of a specific code between the index date and the event date (of note, we excluded ICD-10 A, B, C, L, P, V, W, X, Y, and Z disease categories because of their low relevance for cardiovascular and cerebrovascular events). Third, all medication records from the index date to the event date (n=130). The first 3 digits of the ATC codes for medication records were used for feature construction. Regarding the medication records, we used both the total number of occurrences of a medication (n=65) and the number of days of a medication (n=65). As we used features based on the total number of occurrences in the medical records (ie, ICD-10 codes for diagnosis and ATC codes for medication), null values in a feature mean no occurrence of the disease or medicine for each patient. Therefore, we replaced null values with zeros (eg, the third patient in Figure 1).

After processing the features, we finally obtained 1385 of them for each patient. All the aggregated input vectors were linearly normalized to the range [0,1]. To investigate the advantage of time series characteristics in a longitudinal dataset for predicting diseases, we also developed algorithms based on a Recursive Neural Network (RNN) which is able to capture temporal patterns present in temporal sequenced data. For prediction models based on RNN, each time steps *t* comprised of all medical codes *c*_1_, *c*_2_, …, *c*_|C|_ was converted to a binary vector *x*_*t*_={0,1}^|C|^, and recent 50, 100, or 150 time steps of the binary vector (ie, hospital visits) were used for prediction.

Before attempting machine learning algorithms, a univariate feature selection was performed to remove irrelevant features to the outcome variable. Based on the chi-square statistics scores, 555 significant features (*P*<.05) out of 1385 features were obtained. To avoid model overfitting, the top 55 and 278 features were selected, which represented 10% and 50% of the number of significant features, respectively. Subsequently, we compared the model performances according to the number of features (ie, 55, 278, and 555, respectively). The detailed information for the top 55 selected features and a list with all the features is in Multimedia Appendix 3.

We developed the models based on 6 common machine learning algorithms and compared the performance of the algorithms for predicting the cardio-cerebrovascular outcome of hypertensive patients: logistic regression (LR), support vector machine (SVM) [17], decision tree (DT) [18], random forest (RF) [19], multilayer perceptron (MLP) [20], and long short-term memory (LSTM) for time series prediction. The hyper-parameters used for training models are in Multimedia Appendix 4.

### Model Evaluation Strategy

The following 3 experiments were used as outcomes: (1) predicting a cardiovascular event, (2) predicting a cerebrovascular event, and (3) predicting a cardio-cerebrovascular event (ie, vascular disease). Furthermore, we compared the performances of each algorithm using F1-score according to the number of features. We used 4 evaluation metrics commonly used in classification tasks to evaluate the performance of our models as follows:

- Accuracy: the proportion of patients who were predicted as their actual status
- Precision: the proportion of patients that actually had the diseases out of patients that were predicted as having diseases
- Recall: the proportion of patients that were predicted as having diseases out of all patients who actually had the diseases
- F1-score: the harmonic mean of precision and recall

**Figure 2:**
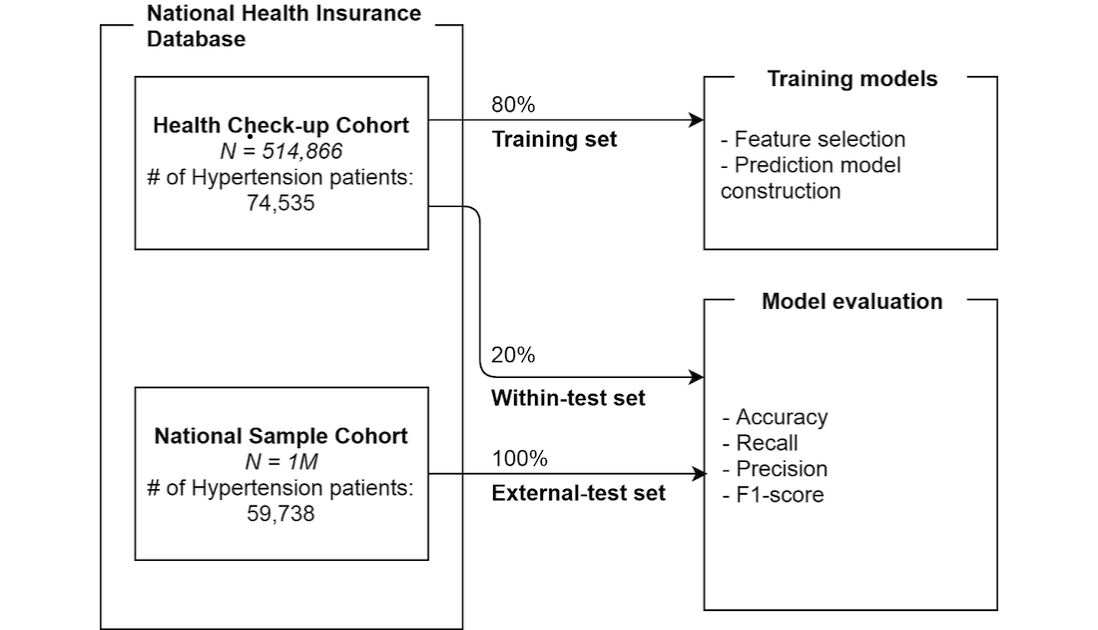
Flowchart of the evaluation strategy.

To compare the evaluation metrics of each machine learning algorithm, the study population extracted from the Health check-up cohort was randomly split into 80% training and 20% test sets. Based on the 80% training set, key features were selected and the prediction models were trained (see Figure 2). We then tested our prediction models using the 20 % test sets (termed within test). After that, to confirm the external validity of the prediction models, the entire study population of the National Sample Cohort was used for external evaluation (termed external test). The training set was once more randomly split into 10-fold to conduct stratified 10-fold cross-validation. The 10-evaluation metrics from the folds were averaged to produce a single estimation.

## Results

### Study Population

**Figure 3:**
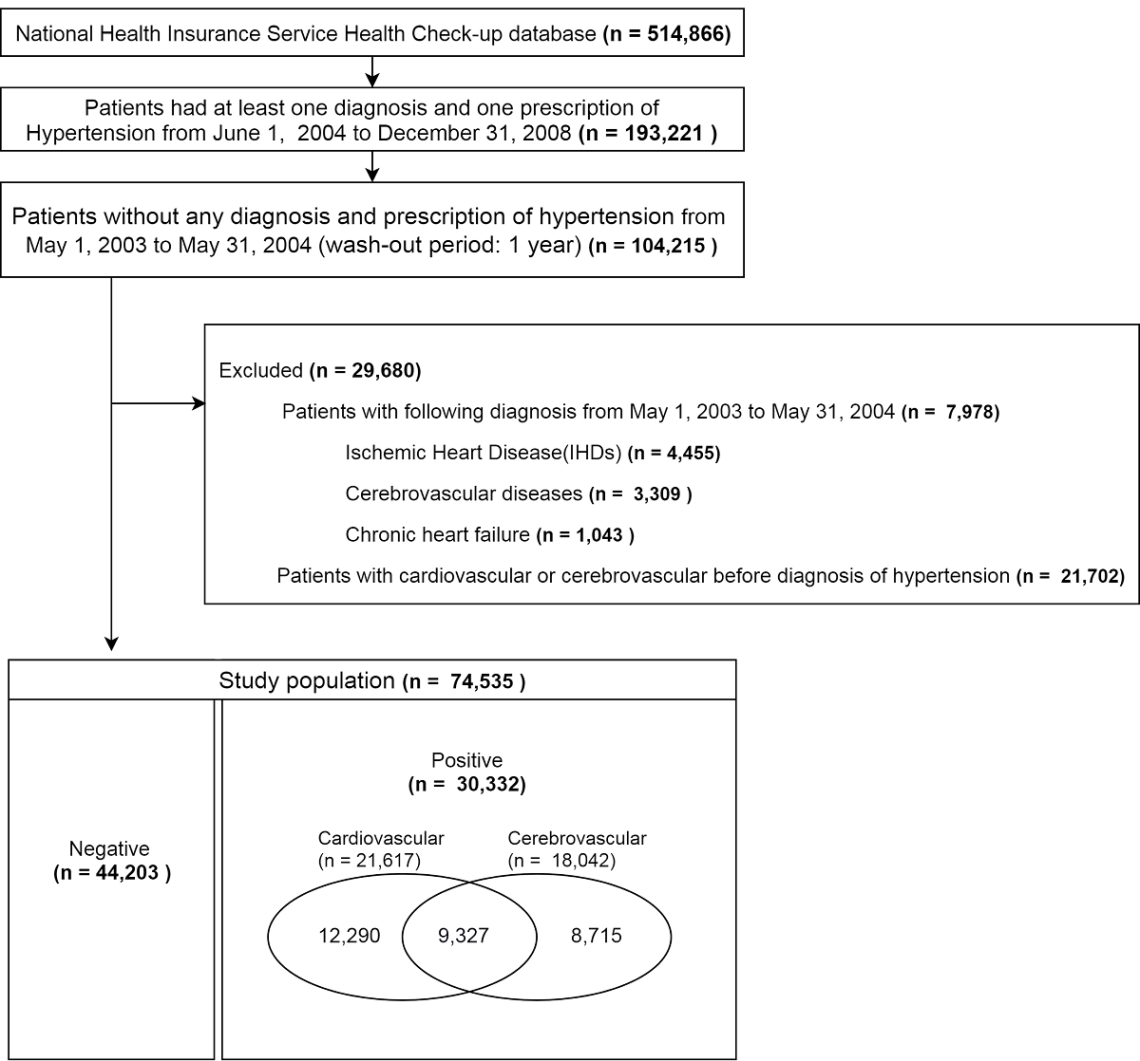
Flowchart describing the study population.

On the basis of the subject definition, we obtained the data of 74,535 patients from the first dataset, for whom a total of 136,843,003 medical records over 12 years were retrieved. As shown in Figure 3, of all hypertensive patients 59% (44,203/74,535) were diagnosed with hypertension only. Among the patients with hypertension, 29% (21,617/74,535) and 24% (18,042/74,535) were diagnosed with cardiovascular and cerebrovascular diseases, respectively.

For the external test, we extracted the data of 67,696 patients with hypertension from the second dataset. To match the age distribution, we only considered patients aged over 40 years. Finally, we obtained the data of 59,738 patients with hypertension. Of all hypertensive patients from the external test set, 60% (36,248/59,738) were diagnosed with hypertension only. Among the patients with hypertension, 28% (16,605/59,738) and 23% (13,828/59,738) were diagnosed with cardiovascular and cerebrovascular diseases, respectively. The disease outcomes were similarly distributed between the within test and external test set. Further characteristics of patients with hypertension only and cardiovascular and cerebrovascular events that were used in training models are presented in Table 2. The variables in the table present patient data at the time point of the first hypertension diagnosis and the patients were grouped by outcome events. The most characteristics were similarly distributed among the groups.

### Performance of Cardio-Cerebrovascular Prediction Models

Table 3 presents the performance of the algorithms with 555 significant features predicting our target outcomes. The results showed that the LSTM algorithm outperforms the other algorithms, except for the recall score in within test, resulting in an accuracy score of .831. While the MLP, LR, and SVM algorithms outperformed RF algorithm in within test with F1 scores of .771, .762, and .760 respectively, the MLP, LR, and SVM algorithms performance in external test dropped dramatically to .065, .013, and .019 respectively, decreasing the recall score to ≤.034. However, DT, RF, and LSTM algorithms showed relatively small performance degradation in the external test. Therefore, in terms of a generalization of the model, the RF algorithm performed best by achieving an F1-score of .705 in the external test for all disease outcomes. The results confirmed that the RF algorithm is the most robust algorithm against overfitting [19,21]. In addition, the results showed the importance of the external validity of disease prediction models. Even though the performance of a model was reliable in within test, we confirmed that it can be vulnerable to new patients from the external test dataset. Therefore, as Damen et al [11] argued, the future research directions of disease prediction should focus on external validation.

The results of each outcome presented in Table 3 show that predicting a single outcome (ie, cardiovascular or cerebrovascular event) showed poor performance compared to predicting the cardio-cerebrovascular events of 5 algorithms. Specifically, predicting cerebrovascular events showed higher performance degradation than predicting cardiovascular events. This fact implies that it could be a good strategy to develop machine learning models predicting similar disease groups as a single outcome, such as cardio-cerebrovascular events in this study. This method will help balance the labels that are mostly imbalanced disease outcomes, and will finally improve the performance of the models as shown by the results of this study.

**Table 2:** General characteristics of the study population.

Variable		Hypertension only (n=44,203)	Cardio-cerebrovascular (n=30,332)	Cardiovascular (n=21,617)	Cerebrovascular (n=18,042)
Age (years), mean (SD)		57.1 (9.1)	60.5 (9.6)	60.3 (9.5)	61.8 (9.4)
**Gender, n (%)^a^**					
	Female	19,036 (43.1)	14,253 (47.0)	9911 (45.7)	8933 (49.5)
	Male	25,167 (56.9)	16,079 (53.0)	11,706 (54.3)	9109 (50.5)
Body mass index, mean (SD)		24.5 (2.9)	24.5 (3.0)	24.6 (3.0)	24.4 (3.0)
**Smoking, n (%)^a^**					
	None	29,144 (65.9)	20,645 (68.1)	14,583 (67.5)	12,558 (69.6)
	Past	4035 (9.1)	2453 (8.1)	1799 (8.3)	1326 (7.3)
	Current	8647 (19.6)	5716 (18.8)	4174 (19.3)	3249 (18.0)
**Drinking, n (%)^a^**					
	Nondrinker	23,645 (53.5)	17,825 (58.8)	12,611 (58.3)	10,979 (60.9)
	Drinker	19,647 (44.4)	11,869 (39.1)	8568 (39.6)	6652 (36.9)
Systolic blood pressure, mean (SD)		137.5 (18.6)	137.2 (18.6)	137.2 (18.7)	137.5 (18.6)
Diastolic blood pressure, mean (SD)		85.3 (12.0)	84.6 (11.9)	84.6 (12.0)	84.4 (11.8)
Total cholesterol, mean (SD)		202.2 (38.4)	203.8 (39.5)	204.2 (39.7)	203.7 (39.0)
Fasting blood sugar level, mean (SD)		102.3 (35.1)	104.5 (39.4)	105 (40.8)	105.0 (39.6)
Diabetes, n (%)^a^		2616 (5.9)	2250 (7.4)	1863 (8.6)	1543 (8.6)
Hyperlipidemia, n (%)^a^		3784 (8.6)	3026 (10.0)	2495 (11.5)	1982 (11.0)

^a^The percent of this variable may not add up to 100% due to the missing value.

**Figure 4:**
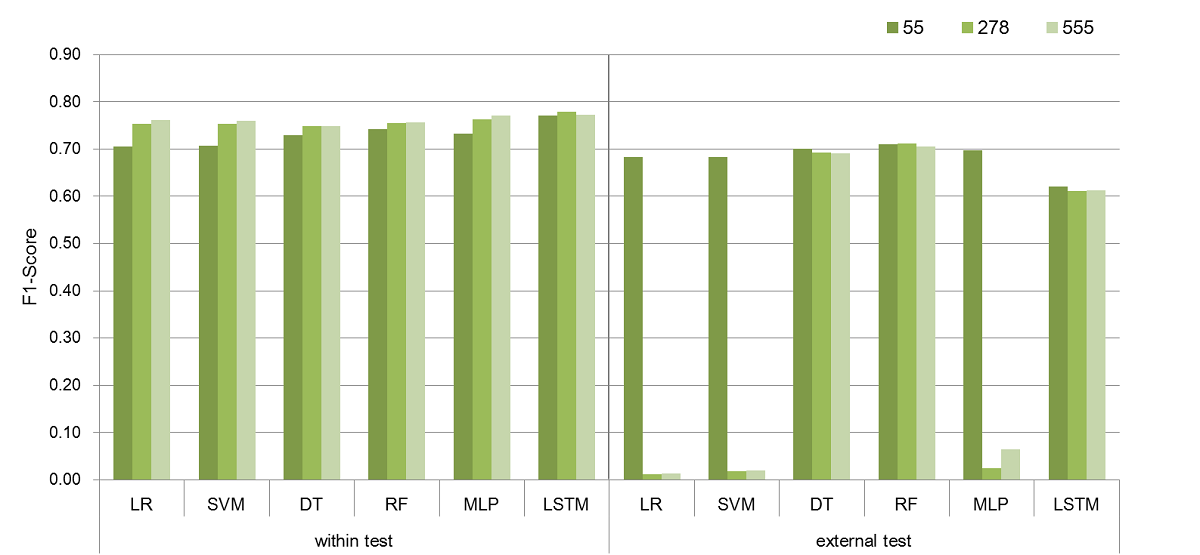
Model evaluation (F1-score) results based on the number of features across 6 models. (LR: logistic regression, SVM: support vector machine, DT: decision tree, RF: random forest, MLP: multilayer perceptron, LSTM: long short-term memory).

Figure 4 shows the F1-scores of each model in both test sets according to the number of features. In the within test, LSTM algorithm outperformed other algorithms, indicating the importance of considering the order of medical records and time information. In the external test, RF algorithm was the best regardless of the number of features. This result clearly indicates that an increasing number of features leads to the model overfitting. For example, while the machine learning algorithms with 55 features showed relatively consistent performance in both within test and external test to predict cardio-cerebrovascular events, the F1-scores of LR, SVM, and MLP algorithms with 278 and 555 features dropped down dramatically even under the same conditions (ie, number of samples, training dataset) except for the number of features. Specifically, the sensitivity of the models was extremely poor (see Table 4). This fact implies that the trained probability threshold does not work properly on new patients’ medical history. Interestingly, RF, DT, and LSTM algorithms showed relatively consistent performance even with changes in the number of features. Therefore, our results indicated that RF is the most robust machine learning algorithm to predict diseases in both external validity and the changes in the number of features.

**Table 3:** Performance of prediction of each outcome across the models with all significant features (N=555).

Prediction outcome algorithms		Within test								External test							
		Accuracy		Recall		Precision		F1-score		Accuracy		Recall		Precision		F1-score	
**Cardio-cerebrovascular**																	
	Logistic regression		.797		.807		.721		.762		.609		.007		.869		.013
	Support vector machine		.796		.803		.722		.760		.610		.009		*.877* ^a^		.019
	Decision tree		.780		*.818*		.691		.749		.740		.737		.650		.691
	Random forest		.793		.799		.718		.757		*.744*		*.779*		.644		*.705*
	Multilayer perceptron		.806		.803		.742		.771		.616		.034		.754		.065
	Long short-term memory		*.831*		.772		*.790*		*.772*		.681		.716		.553		.613
**Cardiovascular**																	
	Logistic regression		.748		.784		.540		.640		.732		.048		*.807*		.091
	Support vector machine		.743		.797		.533		.639		*.735*		.068		.747		.125
	Decision tree		.707		*.814*		.492		.613		.673		*.788*		.449		.572
	Random forest		.723		.798		.509		.622		.685		.787		.461		*.582*
	Multilayer perceptron		*.757*		.782		*.559*		*.652*		.727		.098		.547		.166
**Cerebrovascular**																	
	Logistic regression		.741		.757		.471		.581		*.769*		.002		*.821*		.005
	Support vector machine		.733		.776		.463		.580		.769		.002		.795		.004
	Decision tree		.672		*.828*		.405		.544		.662		.735		.381		.501
	Random forest		.698		.812		.427		.560		.674		*.793*		.397		*.529*
	Multilayer perceptron		*.749*		.787		*.486*		*.601*		.769		.001		.833		.001

^a^The highest scores are presented in italics.

**Table 4:** Prediction for cardio-cerebrovascular according to the number of features across 6 models.

Number of features per algorithms		Within test								External test							
		Accuracy		Recall		Precision		F1-score		Accuracy		Recall		Precision		F1-score	
**55 features**																	
	Logistic regression		.742		.762		.658		.706		.707		.801		.595		.683
	Support vector machine		.742		.764		.657		.707		.707		.803		.594		.683
	Decision tree		.752		*.822* ^a^		.656		.730		.712		.858		.593		.701
	Random forest		.771		.813		.684		.743		*.726*		.858		*.607*		*.711*
	Multilayer perceptron		.766		.778		.691		.732		.722		.815		.610		.698
	Long short-term memory		*.837*		.752		*.810*		*.771*		.618		*.874*		.491		.620
**278 features**																	
	Logistic regression		.788		.793		.717		.753		.609		.006		*.819*		.011
	Support vector machine		.790		.789		.721		.753		.609		.009		.814		.017
	Decision tree		.774		*.828*		.684		.749		.735		.758		.637		.692
	Random forest		.786		.813		.706		.756		*.746*		*.798*		.643		*.712*
	Multilayer perceptron		.802		.785		.742		.763		.609		.012		.690		.024
	Long short-term memory		*.834*		.796		*.778*		*.779*		.653		.770		.521		.611
**555 features**																	
	Logistic regression		.797		.807		.721		.762		.609		.007		*.869*		.013
	Support vector machine		.796		.803		.722		.760		.610		.009		.877		.019
	Decision tree		.780		*.818*		.691		.749		.740		.737		.650		.691
	Random forest		.793		.799		.718		.757		*.744*		*.779*		.644		*.705*
	Multilayer perceptron		.806		.803		.742		.771		.616		.034		.754		.065
	Long short-term memory		*.831*		.772		*.790*		*.772*		.681		.716		.553		.613

^a^The highest scores are presented in italics.

## Discussion

Management of hypertension should be conducted according to the patient’s risk level. In the present study, we developed and compared machine learning models predicting high-risk vascular diseases for hypertensive patients, so that they can manage their blood pressure based on their risk level. The results of this study suggest that machine learning algorithms predict which patients have high risks based on their medical history. To confirm the usefulness of the models that were developed in this study, we conducted the external validation [11,22] using another nationwide claim dataset. The LSTM algorithm outperformed in the within test, and the RF-based algorithm of risk prediction showed better performance over other machine learning algorithms in terms of generalization [21]. The results also confirmed that the models with fewer variables are more generalizable [22].

In recent years, many studies on using machine learning to predict these diseases have been actively conducted with the emergence of large-volume data, such as electronic medical records (EMRs) [23-27]. Previous disease prediction models have used variables from a range of sources, including patient diagnosis and medication, and the models demonstrated better disease predicting performance than more established methods, such as those included in the American Heart Association/American College of Cardiology guidelines [25]. In addition, lab test data and wearable sensor data were also used. Because both these types of data directly reflect the health status of patients, they are very useful for predicting future health complications. However, this fact causes a practical difficulty with using the models, because these data sources involve time-consuming and expensive processes, and not all patients have access to them. Moreover, even with such patient datasets acquired, most developed prediction models are not used in real practice due to the lack of external validity [10-12]. The models were developed by using the datasets of a single institution or of multiple centers and therefore are restricted to predicting the diseases of patients visiting that local site only [22]. We mainly focused on patients’ diagnoses and medication records, so that our models could predict the complications of hypertension with easily obtainable features, and conducted external validation to ensure the generalizability of our models. The prediction model in this study is based on medical history after the hypertension was diagnosed, not a snapshot of the patient’s health status; therefore, patients may obtain their risk levels more appropriately and in a more cost-effective way.

The univariate feature selection method allowed for the improved prediction of outcomes, without overfitting problem. Interestingly, among the top 55 (or 10%) of significant features, a total of 80% of features were related to medication. The result of this feature selection suggests that medication information plays a major role in predicting cardio-cerebrovascular events in hypertensive patients. These results may be related to the fact that the initial treatment may vary according to the patient’s status and clinical decisions [28,29], and medication switching is more frequent than diagnosis switching. Therefore, medication information contains more various and complex information about hypertensive patients compared to diagnostic information [30]. Furthermore, we found a difference between patient groups according to outcome events. Patients without cardio-cerebrovascular outcome events had more prescriptions and medication days. As this study was focused on patient-level prediction through the development and comparison of machine learning algorithms and not on population-level estimations, we did not investigate these treatment patterns in detail. However, this may be an interesting future target for research. We have, therefore, reported the average and median values of top 55 features for the groups in Multimedia Appendix 3.

Moreover, we manually investigated the selected top 55 feature list and found that a considerable number of features seemed irrelevant. Even though the variables seemed not directly related to cardio-cerebrovascular events, we have not removed the features from the model because (1) the variables were selected based on the 12-year treatment characteristics of half million Korean patients and could be associated with omitted variables and (2) the developed model will be applied to Korean patients with the same medical care behavior characteristics. This result provides future research insight investigating the relationship between the target outcome and those uncovered important diagnoses or medications so far based on population-level research using various analysis methods, such as statistical analysis and network analysis [31,32].

We used nationwide claims data including each patient’s treatment and medication history for 12 years to train the machine learning algorithms. There are some limitations in using claims data for predicting diseases. First, there is an issue of the accuracy of disease code due to the purpose of billing [33]. Second, it is difficult to identify the uncovered services and the use of generic medicines that are not prescribed by health care providers. Finally, as we defined clinical events using diagnostic codes, the rates of the event could be underestimated [34].

Despite these limitations, the nationwide claims dataset provides good opportunities to apply the models. First, the dataset contains medical records from almost all medical services (ie, national level), not from a single institution or multiple centers. Furthermore, for scalability of the models, only the diagnosis and medication records of patients were used for training the models. Therefore, the pretrained machine learning algorithm could be used for developing prediction models of extending diseases and improving other prediction models by transferring knowledge, as suggested by Choi et al [35]. Second, the developed model could be applied to manage national-level disease risk. This study is, to the best of our knowledge, the first disease prediction model for patients based on nationwide claims data. Therefore, by relying on the prediction model, a government can predict high-risk patients at a nationwide level and establish health care policies in advance.

There are several future research directions. First, we can refine the models using up-to-date nationwide health insurance data and obtain feedback from physicians about the feasibility and predictability of the models at every single clinic. Second, clinical trials on the effect of controlling hypertension can be conducted by applying dynamic prediction models to patients in every single visit.

## Appendix

Multimedia Appendix 1 Details of the dataset.

Multimedia Appendix 2 Anatomical therapeutic chemical codes for subject definition.

Multimedia Appendix 3 List of features.

Multimedia Appendix 4 Hyper-parameters used for training models.

## Abbreviations

ATC: Anatomical Therapeutic Chemical
DT: decision tree
EMR: electronic medical records
HRA: health risk appraisal
ICD-10: International Classification of Diseases, Tenth Revision
LR: logistic regression
LSTM: long short-term memory
MP: multilayer perceptron
NHIS: National Health Insurance Service
RNN: Recursive Neural Network
RF: random forest
SVM: support vector machine
