# Supplementary material for: Patient-Level Prediction of Cardio-Cerebrovascular Events in Hypertension Using Nationwide Claims Data
Source: J Med Internet Res. 2019 Feb 15;21(2):e11757. doi: 10.2196/11757 (PMC6396076; doi:10.2196/11757)
Supplement: Multimedia Appendix 1 [file jmir_v21i2e11757_app1.pdf]

Multimedia Appendix 1. Details of Dataset

Table S1. Details of dataset

		Sample Cohort DB [1]	Medical Check-up cohort DB [2]
Details	Sample standard	Qualified individuals as of 2002	Qualified individuals as of 2002 in the age of 40-79 in 2002-2003 who received general medical check-up
	Duration	2002-2013 (12 years)	2002-2013 (12 years)
Variables	Contents	- Social & economic qualification variables (including death and disability)<br>- Status of medical resource utilization<br>- Status of clinic	
	Qualification	Health insurance subscribers and Medicare recipients (excluding foreigners)<br>- Total of 14 variables including gender, age, location, type of subscription, social economic variable of the subject such as income rank, disability, death, and etc.	
	Treatment	Payment data to the clinic upon and treatment of subjects at the clinic<br>- Total of 57 variables including date, expenses of treatment, common statement, treatment, type of disease (ICD-10), and prescription: 28 variables in statement, 13 variables in details of treatment, 5 variables in type of disease, and 11 variables in details of prescription.	
	Medical check-up	Major results from medical check-up and behavior and habitual data from questionnaire. Including primary general medical check-up data and transition period check-up data from 2008<br>- Total of 37 variables in 2002-2008 and 41 variables in 2009-2013<br>- Including BMI, systolic blood pressure, diastolic blood pressure, fasting blood sugar level, total cholesterol, smoking, drinking, and etc.	
	Clinic	Status, facility, equipment, and personnel data of clinics by type, establishment, and location (city and state).<br>- Total 10 variables	

References

[1] Details of DB and cost: Sample Cohort DB: National Health Insurance Service 2014 [cited 2018]. URL: https://nhiss.nhis.or.kr/bd/ab/bdaba022eng.do. Archived at: http://www.webcitation.org/71DHtmfsp.

[2] Details of DB and cost: Medical check-up cohort DB: National Health Insurance Service 2014 [cited 2018]. URL: https://nhiss.nhis.or.kr/bd/ab/bdaba022Heng.do. Archived at: http://www.webcitation.org/73WT9ejjw.
