# Supplementary material for: Patient-Level Prediction of Cardio-Cerebrovascular Events in Hypertension Using Nationwide Claims Data
Source: J Med Internet Res. 2019 Feb 15;21(2):e11757. doi: 10.2196/11757 (PMC6396076; doi:10.2196/11757)
Supplement: Multimedia Appendix 2 [file jmir_v21i2e11757_app2.pdf]

Multimedia Appendix 2. Anatomical therapeutic chemical codes for subject definition.

Table S2 The list of ATC Codes for subject definition

Hypertension				Cardiovascular
C07AG	C08CA04	C09AA03	C09DB06	B01AC04
C07AG02	C08CA05	C09AA13	C09DB02	B01AC24
C07AG01	C08DA01	C09BA04	C09DX03	B01AC22
C02CA04	C08CA12	C09AA15	C09DB04	
C01CA17	C08CA15	C09CA06	C09DB01	
G04CA02	C08CA14	C09CA02	C09DB	
G04CA01	C08CA	C09CA	C03DB01	
G04CA	C08CA02	C09CA04	C03CA01	
G04CA04	C09BB05	C09CA01	C03AA03	
G04CA03	C08CA09	C09CA08	C03DA01	
C07AB03	C08CA13	C09CA07	C03CA04	
C07AB07	C08CA11	C09CA03	C03BA04	
C07AB09	C08CA07	C09DA06	C03EA01	
C07AB02	C09AA01	C09DA02	C03BA11	
C07AB12	C09AA02	C09DA		
C07AA05	C09AA16	C09DA04		
C07AA07	C09AA04	C09DA01		
C07AB05	C09AA05	C09DA08		
C08CA01	C09AA	C09DA07		
C08DB01	C09AA08	C09DA03		
