# Supplementary material for: Patient-Level Prediction of Cardio-Cerebrovascular Events in Hypertension Using Nationwide Claims Data
Source: J Med Internet Res. 2019 Feb 15;21(2):e11757. doi: 10.2196/11757 (PMC6396076; doi:10.2196/11757)
Supplement: Multimedia Appendix 3 [file jmir_v21i2e11757_app3.pdf]

Multimedia Appendix 3. List of features.

Table S3. The average and median values of top 55 features for the groups.

			Patients with hypertension only			Patients with cardio-, cerebrovascular outcome		
	Features	Description	median	mean	SD	median	mean	SD
1	ATC_C09	Agents acting on the renin–angiotensin system	7	22.1	29.3	1	7.2	13.9
2	ATC_M09	Other drugs for disorders of the musculo-skeletal system	9	13.9	17.7	2	6.3	11.7
3	I10	Essential Hypertension	34	38.4	36.5	5	12.7	17.4
4	ATC_C09_ days	Agents acting on the renin–angiotensin system	208	778	962.7	6	221.1	424.7
5	ATC_M09_ days	Other drugs for disorders of the musculo-skeletal system	29	56.4	95.8	7	23.9	55.3
6	ATC_R07_ days	Other respiratory system products	0	1.5	7	0	0.2	3.4
7	ATC_R07	Other respiratory system products	0	0.4	1.4	0	0	0.4
8	ATC_C10_ days	Lipid modifying agents	0	348.9	681.9	0	105	292.6
9	ATC_C10	Lipid modifying agents	0	9.8	20.2	0	3.4	9.8
10	J20	Acute bronchitis	1	3.7	6.7	0	1.6	3.6
11	ATC_B01	Antithrombotic agents	0	10.7	21	0	4	10.7
12	ATC_B01_ days	Antithrombotic agents	0	365.1	695	0	120.3	322.7
13	ATC_R06	Antihistamines for systemic use	6	28.3	42.6	1	15	28.7
14	ATC_C08	Agents acting on the renin–angiotensin system	11	26.9	33.1	3	10.9	17
15	ATC_R06_ days	Antihistamines for systemic use	49	120.9	282	18	63.7	177.5
16	ATC_H02	Corticosteroids for systemic use	1	12.1	20.4	0.0	6.7	13.2
17	ATC_S01	Ophthalmologicals	0	9.3	20.1	0.0	5.1	12.5
18	ATC_C04_ days	Peripheral vasodilators	0	71.2	251.8	0.0	28.0	118.4
19	K21	Gastro-esophageal reflux disease	0	1.6	4.9	0.0	0.7	2.7
20	ATC_C08_ days	Agents acting on the renin–angiotensin system	331	896.1	1034.0	45.0	314.8	488.3
21	ATC_H02_ days	Corticosteroids for systemic use	3	51.5	199.2	0.0	26.9	106.8
22	ATC_J01	Antibacterials for systemic use	7	24.1	28.4	2.0	14.5	21.2
23	J30	Vasomotor and allergic rhinitis	0	1.3	4.0	0.0	0.5	2.2

24	ATC_R01_ days	Nasal preparations	0	16.1	44.2	0.0	8.9	28.8
25	ATC_C04	Peripheral vasodilators	0	3.2	10.6	0.0	1.5	5.1
26	ATC_D07	Corticosteroids, dermatological preparations	0	3.2	9.1	0.0	1.7	5.7
27	ATC_R05	Cough and cold preparations	5	27.6	38.7	1.0	16.5	29.0
28	ATC_S01_ days	Ophthalmologicals	0	30.0	119.7	0.0	13.2	49.9
29	ATC_J02_ days	Antimycotics for systemic use	0	9.6	29.8	0.0	4.5	17.4
30	ATC_G04_ days	Urologicals	0	99.5	458.0	0.0	43.5	237.1
31	ATC_M01_ days	Anti-inflammatory and antirheumatic products	126	301.9	523.2	65	184.4	346.5
32	ATC_D06	Antibiotics and chemotherapeutics for dermatological use	0	0.6	1.7	0	0.3	1.2
33	M54	Dorsalgia	1	7.0	24.4	0	3.7	14.2
34	ATC_A05	Bile and liver therapy	0	4.1	14.2	0	2.4	10.2
35	H10		0	1.2	3.5	0	0.6	2.3
36	ATC_M03	Muscle relaxants	6	13.9	25.6	3	9.8	20.0
37	ATC_A03_ days	Drugs for functional gastrointestinal disorders	139	322.2	542.4	73	210.5	393.2
38	ATC_R03_ days	Drugs for obstructive airway diseases	4	57.3	351.0	0	41.3	245.7
39	ATC_B02	Antihemorrhagics	0	0.6	2.0	0	0.3	1.5
40	ATC_D06_ days	Antibiotics and chemotherapeutics for dermatological use	0	0.8	3.2	0	0.4	2.3
41	ATC_A12	Mineral supplements	0	1.8	7.5	0	0.8	4.1
42	M17	Osteoarthritis of knee	0	6.8	22.1	0	3.5	12.6
43	J01	Acute sinusitis	0	0.6	2.6	0	0.2	1.3
44	K29	Gastritis and duodenitis	1	2.5	6.1	0	1.5	4.1
45	H04	Disorders of lacrimal system	0	1.1	3.5	0	0.5	2.2
46	M77	Other enthesopathies	0	0.7	3.8	0	0.2	2.1
47	J40	Bronchitis, not specified as acute or chronic	0	0.63	2.7	0	0.2	1.4
48	ATC_J05_ days	Antivirals for systemic use	0	14.9	168.0	0	4.2	58.7
49	ATC_A07_ days	Antidiarrheals, intestinal anti-inflammatory/anti-infective agents	7	39.1	154.7	3	26.0	101.8
50	S33	Dislocation and sprain of joints and ligaments of lumbar spine and pelvis	0	1.3	5.9	0	0.7	4.7
51	AGE	Age	56	57	9.1	60	60.4	9.5

52	S61	Open wound of wrist, hand and fingers	0	0.3	1.5	0	0.1	0.8
53	J02	Acute pharyngitis	0	1.0	2.9	0	0.5	1.9
54	ATC_J05	Antivirals for systemic use	0	1	4.5	0	0.5	2.2
55	ATC_R03	Drugs for obstructive airway diseases	2	7.6	30.1	0	5.5	22.2

Note: All features beginning with ATC are related to the medication, and the features containing 'days' at the end refer to the feature for the number of days of a medication.

Table S4. Significant feature list

Rank	Feature	Rank	Feature	Rank	Feature	Rank	Feature	Rank	Feature
56	ATC_M01_days	156	ATC_A11_days	256	ATC_G03_days	356	D22	456	N81
57	ATC_D06	157	ATC_M02_days	257	T14	357	ATC_N04_days	457	D30
58	M54	158	J36	258	N60	358	N93	458	N89
59	ATC_A05	159	D23	259	ATC_S02_days	359	D64	459	M85
60	H10	160	S23	260	D21	360	N48	460	S97
61	ATC_M03	161	I83	261	K05	361	R35	461	R59
62	ATC_A03_days	162	M72	262	R07	362	R87	462	G24
63	ATC_R03_days	163	ATC_A11	263	T25	363	K57	463	H53
64	ATC_B02	164	ATC_M04_days	264	J45	364	U23	464	J84
65	ATC_D06_days	165	M53	265	R52	365	S02	465	E55
66	ATC_A12	166	R73	266	E05	366	H18	466	S64
67	M17	167	ATC_A01_days	267	R11	367	ATC_C02	467	M40
68	J01	168	S80	268	H90	368	H92	468	S68
69	K29	169	J31	269	J98	369	E06	469	H47
70	H04	170	ATC_N03	270	H91	370	G25	470	S44
71	M77	171	ATC_B06	271	H93	371	S09	471	R98
72	J40	172	J37	272	N84	372	D14	472	T59
73	ATC_J05_days	173	M23	273	T22	373	F43	473	U50
74	ATC_A07_days	174	H01	274	S66	374	M80	474	R41
75	S33	175	N64	275	S51	375	H34	475	M41
76	AGE	176	S60	276	H68	376	T20	476	INPATIENT
77	S61	177	D34	277	E14	377	K83	477	D15
78	J02	178	J38	278	T63	378	ATC_A04_days	478	T79
79	ATC_J05	179	ATC_M04	279	ATC_J04_days	379	S76	479	Q76
80	ATC_R03	180	D13	280	ATC_P01_days	380	D69	480	H05
81	M48	181	G56	281	K75	381	N73	481	T60
82	ATC_B02_days	182	D17	282	H19	382	S72	482	J47
83	ATC_V03_days	183	J39	283	M60	383	K74	483	G55
84	ATC_A07	184	K30	284	K35	384	R96	484	T75

85	D12	185	ATC_M02	285	M22	385	R68	485	H44
86	M25	186	ATC_C05	286	J15	386	R00	486	J46
87	ATC_M05	187	ATC_A16	287	T24	387	H62	487	D06
88	N40	188	T17	288	G57	388	U24	488	M05
89	J04	189	S05	289	S86	389	R80	489	J80
90	J00	190	E03	290	H57	390	N42	490	H21
91	H00	191	M67	291	R63	391	K70	491	N91
92	S63	192	H43	292	F00	392	R17	492	T33
93	H52	193	N20	293	R42	393	ATC_L04	493	M12
94	S93	194	S92	294	K92	394	S06	494	D01
95	H16	195	J35	295	ATC_P01	395	S41	495	N80
96	ATC_C07	196	ATC_R02	296	R92	396	J05	496	D48
97	H60	197	H65	297	ATC_A16_days	397	K61	497	M46
98	ATC_L01	198	H66	298	ATC_J04	398	D05	498	S65
99	I84	199	R05	299	U30	399	N28	499	N07
100	M51	200	N95	300	J09	400	S96	500	O86
101	K58	201	N39	301	S30	401	D18	501	D11
102	M19	202	S81	302	T78	402	K13	502	R09
103	ATC_L02_days	203	ATC_B03	303	S32	403	ATC_A09	503	U62
104	J32	204	K12	304	R12	404	T18	504	K07
105	H11	205	R91	305	K80	405	H36	505	R43
106	K59	206	G53	306	S70	406	R20	506	E83
107	ATC_C03	207	H61	307	N63	407	R72	507	M61
108	ATC_L01_days	208	R19	308	ATC_A04	408	F32	508	M93
109	S01	209	R31	309	I73	409	I70	509	D33
110	ATC_M05_days	210	M43	310	M20	410	N83	510	G31
111	M50	211	K25	311	E13	411	N13	511	R74
112	S43	212	ATC_C01	312	F41	412	K71	512	K27
113	J22	213	R94	313	J33	413	H69	513	E88
114	ATC_G01	214	S22	314	H73	414	N86	514	G81
115	H25	215	SEX	315	H02	415	M96	515	I88
116	ATC_C07_days	216	D24	316	E58	416	R33	516	Q61
117	ATC_B06_days	217	ATC_D02	317	H20	417	T30	517	E02
118	N30	218	S53	318	R99	418	T31	518	T54
119	J21	219	N41	319	S50	419	ATC_D11_days	519	S57
120	S83	220	M24	320	N45	420	R81	520	K20
121	ATC_N05	221	S00	321	K11	421	F17	521	U78
122	ATC_C03_days	222	N31	322	R93	422	E12	522	Q18
123	H35	223	M71	323	H33	423	D35	523	D31
124	E04	224	S91	324	S21	424	T69	524	G58
125	ATC_N07	225	ATC_R02_days	325	T81	425	N32	525	N43

126	ATC_N07_days	226	M10	326	ATC_C02_days	426	ATC_A09_days	526	N22
127	ATC_H03_days	227	T16	327	S42	427	D27	527	H54
128	E11	228	H81	328	I87	428	K86	528	J67
129	N76	229	S62	329	K82	429	T01	529	E86
130	M81	230	J18	330	S67	430	K56	530	D26
131	R10	231	R51	331	K14	431	N10	531	J42
132	ATC_H01_days	232	E07	332	K81	432	M89	532	G46
133	S46	233	S82	333	K26	433	ATC_D11	533	G43
134	ATC_N05_days	234	J41	334	N21	434	E89	534	N12
135	ATC_A10	235	N72	335	D37	435	H17	535	R26
136	ATC_A01	236	S90	336	M99	436	N02	536	U65
137	ATC_B03_days	237	D50	337	S40	437	F06	537	M16
138	S13	238	ATC_D02_days	338	R03	438	M21	538	U71
139	M47	239	M70	339	R60	439	D36	539	K31
140	J34	240	K62	340	N85	440	N77	540	S54
141	ATC_L02	241	T23	341	N71	441	OUTPATIENT	541	F38
142	ATC_A10_days	242	R22	342	I15	442	S31	542	R76
143	K76	243	R04	343	ATC_N04	443	J11	543	D38
144	H40	244	ATC_G03	344	ATC_L04_days	444	M00	544	R82
145	ATC_C05_days	245	K60	345	I46	445	R25	545	E00
146	K52	246	D25	346	S71	446	S03	546	F89
147	ATC_H03	247	N34	347	I11	447	N75	547	EMERGENCY
148	ATC_G01_days	248	ATC_S02	348	M66	448	F33	548	Q45
149	M62	249	S73	349	T00	449	R55	549	S10
150	T15	250	N87	350	H15	450	ATC_C01_days	550	D16
151	H26	251	F51	351	G51	451	K87	551	I13
152	ATC_H01	252	K40	352	I61	452	T92	552	R23
153	M13	253	S20	353	R50	453	R30	553	D75
154	S52	254	G47	354	D10	454	H72	554	K55
155	ATC_N03_days	255	M76	355	T21	455	T84	555	H06

Note: All features beginning with ATC are related to the medication, and the features containing ‘days’ at the end refer to the feature for the number of days of a medication.
