# Supplementary material for: Patient-Level Prediction of Cardio-Cerebrovascular Events in Hypertension Using Nationwide Claims Data
Source: J Med Internet Res. 2019 Feb 15;21(2):e11757. doi: 10.2196/11757 (PMC6396076; doi:10.2196/11757)
Supplement: Multimedia Appendix 4 [file jmir_v21i2e11757_app4.pdf]

**Multimedia Appendix 4. Hyper-parameters used for training models.**

After experimenting with various values, the following hyper-parameter setting produced the best performance. We implemented algorithms with Keras for LSTM and Scikit-Learn for Logistic regression, SVM, Decision Tree, Random Forest, and MLP.

**Table S5.** Hyper-parameter settings for training the models

| Model | Scikit-Learn/Keras | Hyper-parameter |
|---|---|---|
| Logistic regression | LogisticRegression | L2 regularization: 1.0 |
| SVM | LinearSVC | loss:hinge, Max epoch: 100 |
| Decision Tree | DecisionTreeClassifier | max_depth: 10 |
| Random Forest | RandomForestClassifier | Max_depth: 10, n_estimators:100 |
| MLP with 55 features | MLPClassifier | Activation function: ReLU, Early_stopping:True, Hidden_layer_sizes:37, max_iter:20, alpha:2 |
| MLP with 278 features | MLPClassifier | Activation function: ReLU, Early_stopping:True, Hidden_layer_sizes:186, max_iter:20, alpha:2 |
| MLP with 555 features | MLPClassifier | Activation function: ReLU, Early_stopping:True, Hidden_layer_sizes:371, max_iter:20, alpha:2 |
| LSTM models with 55 features | CuDNNLSTM | Number of layers: 2, batch_size :128, epochs:15, number of steps:150 |
| LSTM models with 258 features | CuDNNLSTM | Number of layers: 1, batch_size:512, epochs:15, number of steps: 150 |
| LSTM models with 555 features | CuDNNLSTM | Number of layers: 1, batch_size: 64, epochs: 5, number of steps: 100 |
